# Nutrient–Microbiota Co-Regulation of Protein Conversion in Black Soldier Fly Larvae: The Role of Alkali-Soluble Protein and Gut Microbial Communities

**DOI:** 10.3390/insects17080856

**Published:** 2026-08-17

**Authors:** Luyao Qi, Shizhao Xiong, Yuanyuan Wei, Zhengzheng Zhao, Yang Ma, Yan Ju, Kanaji Masakorala, Minmin Cai, Chan Yu

**Affiliations:** 1 School of Life Sciences, Hubei University, Wuhan 430062, China; 2 State Key Laboratory of Agricultural Microbiology, National Engineering Research Centre of Microbial Pesticides, College of Life Science and Technology, Huazhong Agricultural University, Wuhan 430070, China; 3 Department of Botany, Faculty of Science, University of Ruhuna, Matara 81000, Sri Lanka

**Keywords:** black soldier fly larvae, alkali-soluble protein, gut microbiota, co-regulation, sustainable protein production

## Abstract

Insect protein farming offers sustainable advantages in land efficiency, emission reductions, and bioconversion, yet optimizing the nutrient composition remains a major challenge for cost-effective production. This study investigates the co-regulatory mechanism between alkali-soluble protein (SpA) and the gut microbiota in black soldier fly larvae (*Hermetia illucens*) and their effect on protein conversion efficiency. Feeding trials with varying alfalfa/SpA ratios identified a wheat middlings/alfalfa meal blend at a (5:0 ratio) as optimal for promoting larval protein accumulation. SDS-PAGE and 16S rRNA analyses revealed a strong positive correlation between SpA and larval crude protein (R^2^ = 0.82). The network analysis and Pearson correlation heatmap further confirmed positive correlations among SpA, larval protein, *Enterococcus*, and *Ignatzschineria* (*p* < 0.05), suggesting that high SpA in the substrate was associated with the enrichment of these taxa, which synergistically enhanced proteolysis through alkaline protease secretion (R^2^ = 0.85) and chitinase-mediated gut remodeling. Multi-linear regression modeling verified SpA as a superior predictor of the crude protein content compared with total nitrogen (TN), improving the model’s coefficient of determination (R^2^) from 0.40 to 0.82. These findings highlight SpA’s higher bioavailability and its direct role in metabolic utilization. By integrating the feed composition, microbiome function, and host metabolism, this study established a regulatory network driving larval protein biosynthesis. The targeted modulation of dietary SpA content may offer a promising approach to enhance beneficial microbial communities and improve protein conversion efficiency in BSFL-rearing systems. These findings provide a theoretical basis for optimizing feed formulations to support sustainable insect protein production from organic waste.

## Simple Summary

The black soldier fly is an insect that can convert organic waste into high-quality protein, offering a sustainable solution for animal feed production. However, the efficiency of this conversion depends largely on the type of nutrients provided in the feed. In this study, we investigated how different protein components in the feed affect larval growth and protein accumulation, as well as the role of gut bacteria in this process. We found that a specific protein fraction called alkali-soluble protein (SpA) was the most important factor driving protein accumulation in the larvae. Larvae fed diets rich in SpA grew larger and produced more protein. We also identified two groups of gut bacteria that were closely linked to improved protein conversion. These findings suggest that by carefully selecting feed ingredients with a high SpA content, we can significantly enhance protein production from black soldier fly larvae. This research provides practical guidance for optimizing feed formulations, contributing to more sustainable food systems and reducing reliance on traditional protein sources such as soybean meal.

## 1. Introduction

The global population is projected to approach 10 billion by 2025 driving a substantial increase in food demand [1]. Rising living standards and shifting dietary habits are accelerating the expansion of large-scale animal husbandry, which in turn intensifies the demand for animal feed [2]. This surge has created a severe global shortage of protein resources, highlighting the urgency of identifying alternative protein sources. Among them, the black soldier fly has gained significant international attention as a promising candidate.

The black soldier fly larvae (BSFL), a saprophytic insect species (Linnaeus, 1758; Diptera: Stratiomyidae) [3], is characterized by rapid reproduction, high bioconversion efficiency, strong stress tolerance, and broad dietary adaptability. BSFL contain substantial nutritional value, with crude protein and fat contents ranging from 32% to 52% and 31% to 39%, respectively [4,5,6]. These traits make them an attractive option for animal feed and potentially for human consumption. BSFL powder, as a processed animal protein, has already been applied in aquaculture [7], pet food [8], and livestock feed, including pigs and poultry [9], thereby contributing to alleviating the shortage of protein in animal production. Moreover, compared with conventional livestock farming, black soldier fly cultivation requires less land, water, and energy and generates lower greenhouse gas emissions.

Over the past decade, BSFL have become a model insect for global research because of their nutritional potential [10]. Studies have systematically examined the abiotic factors (e.g., temperature, photoperiod, humidity, and substrate composition) and biotic factors (e.g., substrate-associated and enteric microbial consortia) that influence nutritional profiles [11]. Substrate quality, especially the nitrogen content and the carbon-to-nitrogen (C/N) ratio, is a critical determinant of larval development and nutrient-assimilation efficiency. For instance, Lu et al. [12] reported that the choice of nitrogen source significantly affects the larval biomass yield during food waste valorization. Nevertheless, despite clear associations between dietary nitrogen and insect protein content, the bioavailability of nitrogen forms (e.g., alkali-soluble protein (SpA) vs. acid-soluble protein (SpC)) remains poorly understood. Specifically, whether other nitrogen pools have a higher bioavailability over TN and how it shapes a functional microbiome to enhance nutrient utilization are unexplored questions central to optimizing BSFL as a bioconversion system. Therefore, the important improvements of this study over the current research are threefold: (1) to shift the focus from total nitrogen to the bioavailable SpA fraction as a key dietary determinant; (2) to move beyond parallel observations of diet and microbiota by integrating them into a ‘Substrate–Microbiota–Protein’ co-regulatory framework; and (3) to translate correlations into a predictive model and testable mechanistic hypothesis for future research. Addressing this gap is essential for designing targeted nutritional strategies to fully harness BSFL as a sustainable protein resource.

This study investigates the nutritional regulation of BSFL with a focus on identifying feed-derived nutrients that drive larval growth and protein enrichment, while clarifying the intrinsic interactions between gut microbial communities and protein accumulation dynamics. The objectives are as follows: (I) to characterize the regulatory role of alkali-soluble proteins in feed formulations in BSFL growth performance and protein biosynthesis pathways; (II) deciphering the treatment-specific associations between the BSFL gut microbiota and host protein content by integrating 16S rRNA gene sequencing with host nutritional data; (III) to establish a tripartite framework linking feed components, gut microbiome remodeling, and protein metabolic networks to define key transformation pathways in the BSFL intestinal system; (IV) to propose a nutrient–microbiota co-regulation model for targeted protein enhancement, informed by identified metabolic checkpoints and microbial consortia. This research provides both a theoretical basis for optimizing BSFL protein production and practical guidance for improving organic waste utilization and mitigating the shortage of animal feed protein.

## 2. Materials and Methods

### 2.1. BSFL and Feed

BSFL were mass reared under controlled conditions (27.5 °C, 70% relative humidity) at Huazhong Agricultural University for 4 days on an artificial diet comprising wheat bran and wheat middlings (1:1, *w*/*w*). Uniform 4-day-old larvae (with a similar body size and weight and no visible morphological abnormalities) were used for subsequent experiments.

Experimental feed components included wheat bran and second-grade flour (Huazhong Agricultural University), soybean meal (SBM; Jiahui Feed, Shijiazhuang, China), cottonseed meal (COM; Jiahui Feed), peanut meal (PEM; Ruijun Oil Mill, Zhengzhou, China), alfalfa meal (ALM; Baifa Animal Food Co., Ltd., Yichun, China), soybean straw powder (SSP; Huifeng Straw Products, Lianyungang, China), and foxtal millet husk powder (FHP; Pingshuo Trade, Huainan, China). All materials were oven-dried at 65 °C to a constant weight, pulverized using a grinder (Royalstar, Jinhua, China), and sieved through a 0.5 mm mesh. Physicochemical characteristics are provided in the Supplementary Materials.

### 2.2. Experimental Design

The experiment was conducted with three independent biological replicates per treatment, with each biological replicate consisting of a separate cylindrical container (250 mm diameter × 120 mm height) containing 100 larvae. All containers were maintained under controlled environmental conditions (27.5 °C, 70% relative humidity), and each container was supplied with 50 g of unsterilized artificial diet (70% moisture content) [13]. Containers were randomly arranged on rearing shelves using a complete randomized block design, with the shelf position treated as a blocking factor. Larvae were randomly allocated to each container at the start of the experiment. During feeding, 10 larvae were randomly sampled from each container daily and weighed to monitor growth. After the measurement, the larvae were returned to their respective containers. The experiment was terminated when 10% of larvae entered the prepupal stage or when the larval weight plateaued [14]. The prepupal stage was morphologically defined by a darkened cuticle, shortened body length, and the complete cessation of feeding; the prepupal ratio was determined via the daily inspection of 30 randomly sampled larvae per container. A weight plateau was defined as no significant increase in the average larval fresh weight for two consecutive weighing days. For analytical measurements, three technical subsamples were taken from each biological replicate and averaged prior to statistical analysis. At the end of the experiment, the remaining substrate was weighed, and both the number and weight of surviving larvae were recorded to calculate survival rates.

Three experiments were conducted to investigate the regulation of protein accumulation in BSFL. The compositions of the experimental diets are shown in Supplementary Figure S1. In Experiment 1, key nutritional factors were screened using diets in which wheat bran was fixed at 50%, and the WHM–alfalfa meal (ALM) ratio was varied across a gradient (5:0, 4:1, 3:2, 2:3, 1:4, 0:5). Experiment 2 generalized the role of SpA, identified in Experiment 1, by employing diets with a fixed 1:1 ratio of wheat bran to different agricultural by-products: SBM, PEM, COM, ALM, SSP, and FHP. Experiment 3 isolated the specific effect of SpA by standardizing total SpA intake across treatments, adjusting the absolute substrate quantities from Experiment 2. This validated its causal influence on larval protein biosynthesis.

### 2.3. Physicochemical Analysis

Collected BSFL were analyzed for the moisture, crude protein, and crude fat content. The moisture content was determined using the constant weight method, using an oven at 105 °C for 4 h. The nutritional parameters of the substrates, including organic matter (OM), total phosphorus (TP), total nitrogen (TN), and total potassium (TK), were measured according to the standard NY/T 525-2021 [15] standard (Standardization Administration of China) [16]. Crude protein (CP) was determined using the alkaline potassium permanganate method, with a nitrogen-to-protein conversion factor of 6.25. Crude fat was determined using the Soxhlet extraction method [17].

SpA was measured following GB/T 19541-2017 [18]. Briefly, 0.5 g of the feed sample was homogenized with 25 mL of 0.2% potassium hydroxide solution (2 g of the analytical reagent (AR) potassium hydroxide dissolved in 1000 mL of pure water) and stirred using a magnetic stirrer at 700 rpm for 20 min at room temperature. The mixture was centrifuged at 2700× *g* for 10 min, and the supernatant was collected. SpC was determined according to NY/T 3801-2020 [19]. A 0.5 g feed sample was mixed with 10 mL of 10% trichloroacetic acid (10 g of AR trichloroacetic acid dissolved in 100 mL of pure water), shaken at 150 rpm for 30 min, and centrifuged at 4000× *g* for 5 min to isolate the supernatant. Water-soluble protein (SpW) was determined according NY/T 1205-2006 [20]. A 0.5 g sample was combined with 10 mL of distilled water, shaken at 150 rpm for 60 min at 20 °C, and centrifuged at 2000× *g* for 10 min to collect the supernatant. Pepsin-digested protein (SpP) was determined following GB/T 17811-2008 [21]. A 0.5 g feed sample was digested with 75 mL of freshly prepared pepsin solution (20 IU/mL), at 45 °C for 16 h under constant agitation. The digestate was filtered through a Büchner funnel, and the filtrate was collected for analysis. All supernatants were further analyzed for protein content using the potassium persulfate method [22]. For all spectrophotometric quantifications of total nitrogen, a standard curve was constructed using total nitrogen standards (concentration range: 0–40 µg mL^−1^). The calibration curve yielded the equation y = 0.0243x − 0.0017, with a coefficient of determination R^2^ = 0.9981 (Figure S2), confirming excellent linearity and reliability of the colorimetric assay across the tested concentration range. All samples were measured in triplicate; the relative deviation between replicate measurements was consistently below 5%, indicating acceptable method precision. All samples from different treatment groups were processed in parallel using identical reagent lots and extraction conditions to ensure comparability among treatments. The standard curve for total nitrogen quantification is provided in Figure S2.

### 2.4. SDS-PAGE Analysis

SDS-PAGE analysis was performed for SpA, SpW, SpC, and SpP, following the method described by He et al. [23]. Briefly, 2 mL of each protein supernatant was mixed with 4 mL of acetone and incubated overnight at 4 °C. The mixture was centrifuged at 10,000× *g* for 15 min, and the pellet was air-dried at room temperature and then dissolved in 0.5 mL of ultrapure water. SDS-PAGE was performed using the Omni-Easy™ One-step PAGE Gel Fast Preparation Kit (12.5%, Shanghai Epizyme Biomedical Technology Co., Ltd., Shanghai, China). A 1.0 mm-thick gel was prepared, and 20 µL of each sample was mixed with 5 µL of 5× loading buffer, heated at 90 °C for 15 min, cooled, and centrifuged at 6700 rpm for 1 min. The running buffer (10× Tris/Glycine/SDS buffer diluted to 1×) was used, and electrophoresis was conducted at 200 V for 40 min. After electrophoresis, gels were stained with 50 mL of Coomassie Brilliant Blue R-250 solution (Yeasen Biotechnology Co., Ltd., Shanghai, China) under continuous agitation for 30 min, rinsed in 200 mL deionized water, and destained for 24 h with intermittent changes of the destaining solution [24].

### 2.5. DNA Extraction

At the end of the experiment, larvae were separated from the substrate using a sieve, and five individuals were randomly selected for gut extraction. The sampled larvae were starved for 24 h to empty intestinal contents and minimize the presence of non-symbiotic bacteria. The larvae were then disinfected with 75% ethanol for 2 min and rinsed three times with sterile water. Dissection was performed on an ultra-clean bench under ultraviolet disinfection. Sterilized anatomical scissors were used to open the larvae, and the gut was collected with sterile tweezers. The gut tissue was homogenized, and the grinding solution was centrifuged at 12,000× *g* for 20 min at 4 °C, with the precipitate retained. All intestinal samples were stored at −80 °C until DNA extraction. Total microbial DNA was extracted from gut homogenates using a modified CTAB–phenol–chloroform protocol. Briefly, samples were lysed in CTAB buffer with proteinase K and SDS, followed by phenol–chloroform purification, isopropanol precipitation, and RNase A digestion [25]. Detailed extraction steps are provided in Supplementary Method S1. Negative controls (sterile water without sample) were processed in parallel throughout the extraction procedure and yielded no detectable DNA, confirming the absence of reagent contamination.

### 2.6. 16S rRNA Sequencing

To characterize the gut bacterial community, DNA samples were amplified and sequenced for the 16S rRNA V3–V4 region using the Illumina HiSeq 2500 platform (Shanghai Major Biomedical Technology Co., Ltd., Shanghai, China). PCR amplification employed primers 338F (5′-ACTCCTACGGGAGGCAGCAG-3′) and 806R (5′-GGACTACHVGGGTWTCTAAT-3′). PCR conditions consisted of an initial denaturation at 95 °C for 3 min, followed by 28 cycles of 95 °C for 30 s, 57 °C for 30 s, and 72 °C for 45 s, with a final extension at 72 °C for 10 min, then holding at 10 °C. Raw sequencing data were processed using QIIME2 (v2022.2) with the DADA2 plugin for quality filtering, chimera removal, and paired-end merging. A total of 5,795,594 raw paired-end reads were generated across 21 samples (7 treatments × 3 biological replicates). After quality control, 2,664,056 high-quality sequences remained, with an average of 126,860 sequences per sample and a mean length of 427 bp. Rarefaction curves approached saturation for all samples (Figure S5), confirming that the sequencing depth was sufficient to capture the majority of bacterial diversity. Taxonomy was assigned using the SILVA database (v138) with a 97% similarity threshold. For alpha- and beta-diversity analyses, sequence counts were rarefied to an even depth. Beta-diversity was assessed via PERMANOVA (999 permutations) based on Bray–Curtis distances. Negative controls (sterile water) were included in the PCR step and showed no detectable amplification products. Heatmap analysis and redundancy analysis of the bacterial communities were performed on the Majorbio BioCloud platform (Shanghai Majorbio Bio-Pharm Technology Co., Ltd., Shanghai, China).

### 2.7. Statistical Analysis

One-way analysis of variance (ANOVA) was used to evaluate group differences after confirming normality with Shapiro–Wilk tests and homogeneity of variances with Levene’s test using SPSS 19 (IBM Corporation, New York, NY, USA). Statistical significance was set at *p* < 0.05. For multiple comparisons, Fisher’s LSD and Duncan’s tests were performed; alpha-diversity comparisons were corrected using FDR. Given the exploratory nature of our study, we acknowledge that the use of Fisher’s LSD may inflate Type I error rates, and therefore, we prioritized the consistency of significant results across both LSD and Duncan’s tests as an indicator of robust differences. Multiple comparisons of alpha-diversity indices were corrected using the Benjamini–Hochberg FDR method. The consistency of significant results across both tests was used to support the robustness of the observed differences. To ensure the robustness and reliability of the multiple linear regression models developed in this study, a comprehensive validation procedure was implemented. The variance inflation factor (VIF) was calculated for all predictors within each model to diagnose multicollinearity. A VIF value of less than 5 was taken as an indication of no significant multicollinearity, ensuring the stability of the coefficient estimates [26]. Furthermore, residual analysis was performed by plotting the residuals against the predicted values for key representative models. This was done to verify the critical assumptions of linear regression, namely linearity and homoscedasticity (constant variance of residuals). For the multiple linear regression models developed in this study, comprehensive diagnostics were performed to verify the assumptions of linear regression. Linearity and homoscedasticity were assessed by plotting residuals against fitted values, multicollinearity was evaluated using the variance inflation factor (VIF), and the normality of residuals was examined using Q–Q plots and Shapiro–Wilk tests. To address the uncertainty estimation, standard errors (SEs) for all regression coefficients were calculated.

Correlation heatmaps, linear regression, and other graphical analyses were performed with Origin 2021 (OriginLab Corporation, Northampton, MA, USA). Additional diagrams were constructed using GraphPad Prism 9.4.0 (GraphPad Software, San Diego, CA, USA). All statistical analyses were carried out on the mean values of three biological replicates per treatment. Technical subsamples were averaged for each biological replicate prior to analysis.

## 3. Results

### 3.1. Regulation of Black Soldier Fly Larval Protein by ALM at Different Proportions

**Figure 1:**
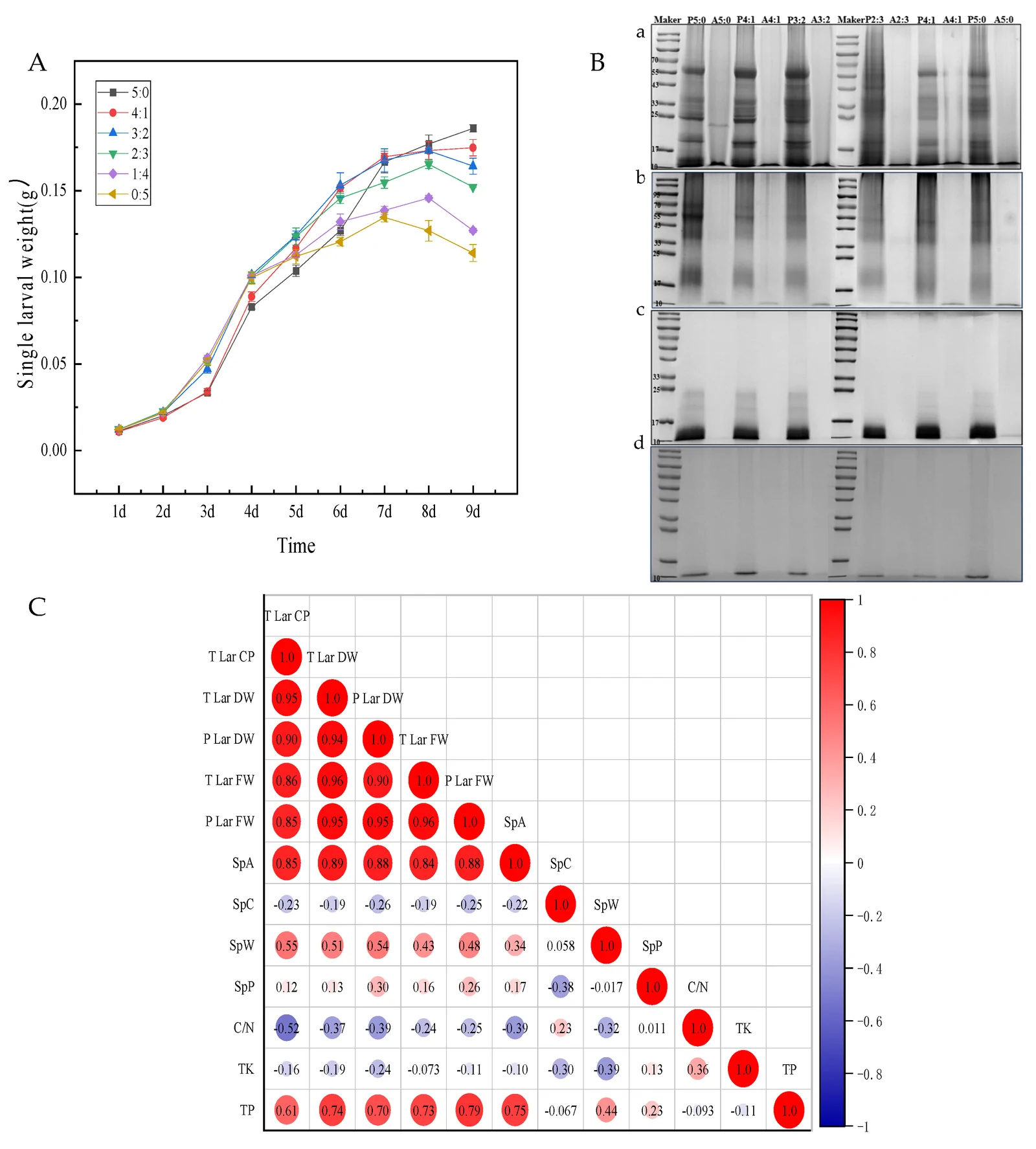
The effects of different proportions of alfalfa meal on the growth of 100 BSFLs. (**A**) The growth of BSFL. (**B**) Protein gel electrophoresis analysis during the feed conversion process: (**a**) SpW; (**b**) SpA; (**c**) SpP; (**d**) SpC; P represents the initial feed, while A represents the final feed treated with BSFLs. (**C**) Pearson correlation heatmap comparing larval growth parameters and feed physicochemical properties (OM, organic matter; TN, total nitrogen; TP, total phosphorus; TK, total potassium; C/N, carbon-to-nitrogen ratio). Error bars represent means ± SDs of three biological replicates (*n* = 3).

Larval growth dynamics under different ALM proportions are shown in Figure 1A. A biphasic growth pattern was observed, with fresh biomass peaking on day 7 and declining by day 8. In Group A (5:0 ratio), crude protein was 2.24 ± 0.1 g, dry weight was 4.2 ± 0.1 g, and fresh weight was 16 ± 0.4 g. By contrast, Group F (0:5 ratio) exhibited significantly lower values (*p* < 0.001): crude protein of 1.17 ± 0.1 g, dry weight of 2.1 ± 0.1 g, and fresh weight of 8.8 ± 0.5 g (Table 1). Overall, the larval crude protein, dry weight, and fresh weight decreased progressively with increasing ALM proportions. For all statistical comparisons, exact *p*-values are reported in the tables (Table 1, Table 2 and Table 3 and Table S2). In the text, *p*-values are presented as *p* < 0.05 or *p* < 0.01 for brevity when multiple comparisons are summarized.

The feed physicochemical properties strongly affected larval growth and nutrient accumulation. Protein gel electrophoresis (Figure 1B) revealed that characteristic protein bands decreased or disappeared after BSFL digestion. Bands corresponding to SpW and SpA showed the most pronounced decline, suggesting higher utilization efficiency of these fractions. Significant intergroup differences were detected in the initial feed SpA and TK content (*p* < 0.05), with Group A containing the highest SpA (10.3% ± 0.4%), compared with Group F (7.3% ± 0.3%). By contrast, the TK and SpC content changed little before and after larval transformation. Correlation heatmap analysis (Figure 1C) showed that the crude protein content and weight gain rate were positively correlated with SpA, TN, and TP levels in the feed (*p* < 0.01), with the strongest correlation observed for SpA (R^2^ = 0.82). Given its superior correlation strength, SpA was consequently identified as the key nutritional factor for focused investigation in subsequent experiments.

### 3.2. Effects of Different SpA Concentrations on BSFL Growth

**Figure 2:**
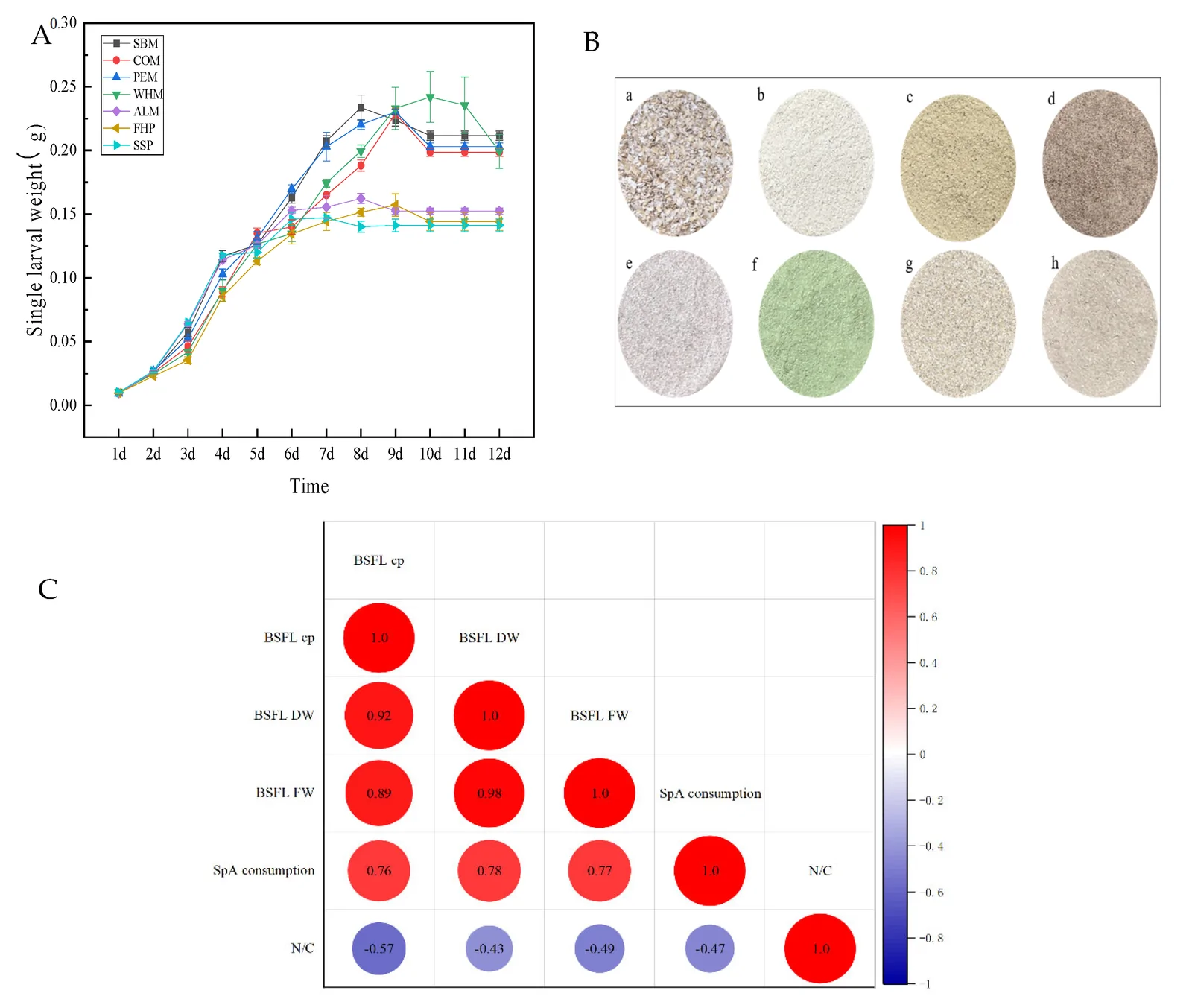
Impact of varying concentrations of SpA in on the growth of BSFL. (**A**) The growth of BSFL. (**B**) Feed: (**a**) wheat bran; (**b**) soybean meal; (**c**) cottonseed meal; (**d**) peanut meal; (**e**) wheat middlings; (**f**) alfalfa meal; (**g**) foxtal millet husk powder; (**h**) soybean straw powder. (**C**) Heatmap of Pearson correlation coefficients between BSFL growth and feed physicochemical properties. Error bars represent means ± SDs of three biological replicates (*n* = 3).

Figure 2A illustrates the effects of the dietary composition on BSFL growth and development. Larvae reared on SBM, COM, PEM, and WHM exhibited significantly higher final weights than those fed ALM, FHP, or SSP (*p* < 0.001). The observed disparity is consistent with the higher fiber levels in ALM, FHP, and SSP diets, compared with the lower fiber and higher protein content in the SBM, COM, PEM, and WHM diets (Figure 2B). As shown in Table 2, larvae reared on high-SpA diets, such as COM (5.1 ± 0.1 g SpA) and WHM (3.6 ± 0.1 g SpA), exhibited superior growth performance and protein accumulation. The crude protein content reached 2.34 ± 0.1 g and 2.39 ± 0.1 g in the COM and WHM groups, respectively, significantly exceeding values in the low-SpA groups such as FHP (1.06 ± 0.0 g) and SSP (1.03 ± 0.1 g). Similarly, dry weight and fresh weight were greatest in the WHM (5.1 ± 0.4 g) and COM (16 ± 1.5 g) groups (*p* < 0.05).

Diets with higher SpA (COM and SBM) showed greater feed uptake (5.1 ± 0.1 g each). Correlation heatmap analysis (Figure 2C) showed that SpA consumption was positively correlated with BSFL CP (R = 0.76), DW (R = 0.78), and FW (R = 0.89). Consumption showed negative correlations with BSFL CP (R = −0.57), DW (R = −0.49), and FW (R = −0.49). C/N also showed negative correlations with BSFL CP (R = −0.43), DW (R = −0.47), and FW (R = −0.47).

### 3.3. Gut Microbiota Diversity in BSFL

**Figure 3:**
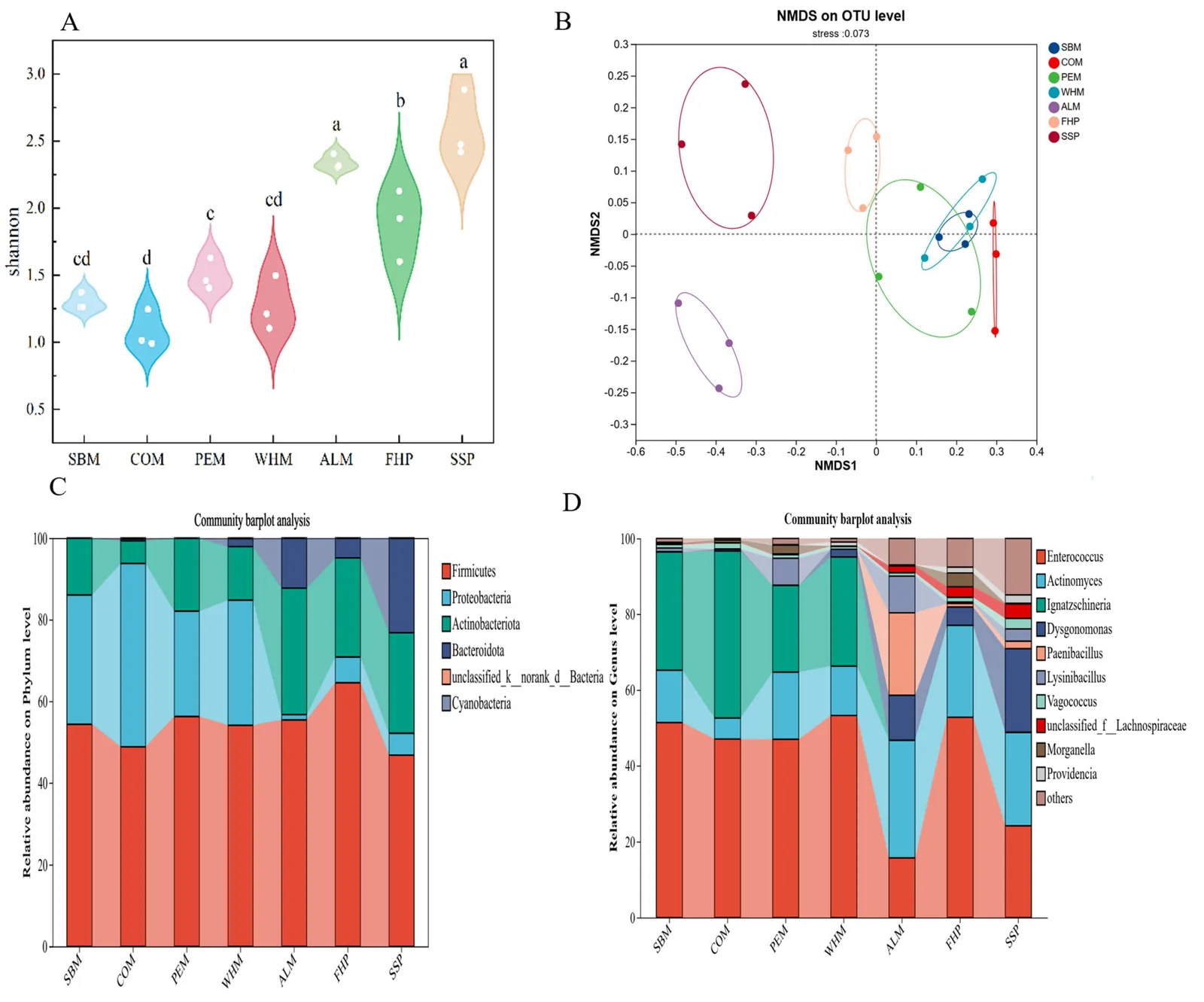
Larval gut bacterial community diversity in different groups. (**A**) Shannon indices at the OTU level (*n* = 3), different letters indicate significant differences (*p* < 0.05). (**B**) Non-metric multidimensional scaling analysis (NMDS) at the OTU level. (**C**) Community diversity at the phylum level. (**D**) Community diversity at the genus level.

This study investigated the diversity of the gut microbiota in BSFL and the relative abundances of different microbial groups across treatment conditions. A total of 2,664,056 high-quality sequences were obtained from the 21 gut samples, with an average of 126,860 sequences per sample. Rarefaction curves approached saturation for all samples (Figure S5), confirming adequate sequencing depth to characterize the bacterial communities. At the operational taxonomic unit (OTU) level, Shannon indices revealed significant differences in microbial α-diversity across dietary treatments (*p* < 0.001; Figure 3A). Larvae fed high-fiber substrates (SSP: 2.6 ± 0.1; ALM: 2.3 ± 0.1) exhibited higher diversity than those in the high-protein groups (COM: 1.1 ± 0.1; WHM: 1.3 ± 0.1). NMDS analysis separated high-fiber (ALM, SSP) from high-protein (COM, WHM) clusters along Axis 1, whereas transitional substrates (SBM, PEM) occupied intermediate positions (Figure 3B).

Hierarchical clustering revealed feed-specific microbiome compositions at both the phylum and genus levels (Figure 3C,D). At the phylum level, Firmicutes was the most abundant phylum across all groups, ranging from 46% to 65%, followed by *Proteobacteria* (25–44%), *Actinobacteriota* (24–31%), and *Bacteroidota* (0–23%). The relative abundance of Firmicutes was higher in COM (65%) and WHM (62%) compared with ALM (46%) and SSP (48%). Proteobacteria abundance was higher in WHM (44%) and PEM (38%) than in ALM (25%) and SSP (28%). *Actinobacteriota* was more abundant in ALM (31%) and SSP (29%) compared with COM (24%) and WHM (25%). *Bacteroidota* was most abundant in SSP (23%), followed by ALM (18%), whereas it was nearly absent in COM (2%) and WHM (3%). At the genus level, *Enterococcus* (16–51%), *Actinomyces* (6–31%), *Ignatzschineria* (0–44%), and *Dysgonomonas* (0–22%) were the predominant genera across treatments. The relative abundance of Enterococcus was highest in COM (51%), followed by WHM (43%) and SBM (38%), and lowest in ALM (16%) and SSP (18%). *Ignatzschineria* abundance was highest in WHM (44%), followed by PEM (35%), and was below 5% in ALM and SSP. Actinomyces showed higher abundance in ALM (31%) and SSP (28%) than in COM (6%) and WHM (8%). *Dysgonomonas* was more abundant in SSP (22%) and ALM (16%) compared with COM (2%) and WHM (3%).

### 3.4. Alkali-Soluble Protein-Regulation Strategies

To clarify the association between dietary components and larval protein biosynthesis, stepwise multiple linear regression was employed to develop models with BSFL CP and DW as dependent variables and feed physicochemical properties as independent variables. For the model validation, we confirmed the absence of significant multicollinearity through VIF analysis (all values < 2) and verified the assumptions of linearity and homoscedasticity via residual analysis. The applicable conditions of the models are defined by the experimental data ranges: OM content of 40–50 g, SpA content of 2–6 g, and the use of common agricultural by-products under controlled rearing conditions (4-day-old larvae, 27.5 °C, 70% relative humidity). Predictions are most reliable within these calibrated boundaries. The model equations were as follows:

Experiment 1 (alfalfa proportion gradient):

$$\mathrm{BSFL}\;\mathrm{CP}\;=\;-2.37193\;+\;0.02294\mathrm{OM}\;+\;1.34847\mathrm{TN}\;(R^{2}\;=\;0.3961);$$

$$\mathrm{BSFL}\;\mathrm{CP}=\;-3.81101+0.04087\mathrm{OM}+1.12047\mathrm{SpA}\;(R^{2}=0.822);$$

$$\mathrm{BSFL}\;\mathrm{DW}=\;-0.3886-0.01353\mathrm{OM}+0.77848\mathrm{TN}\;(R^{2}=0.3622);$$

$$\mathrm{BSFL}\;\mathrm{DW}=\;-1.72878+0.32163\mathrm{OM}+0.49201\mathrm{SpA}\;(R^{2}=0.7491).$$

Experiment 2 (SpA concentration gradient):

$$\mathrm{BSFL}\;\mathrm{CP}\;=\;5.3019\;-\;0.0845\mathrm{OM}\;+\;0.03117\mathrm{TN}\;\left(R^{2}\;=\;0.091\right);$$

$$\mathrm{BSFL}\;\mathrm{CP}=\;-1.38164+0.0366\mathrm{OM}+0.3965\mathrm{SpA}\;(R^{2}=0.556);$$

$$\mathrm{BSFL}\;\mathrm{DW}=13.31184+0.01276\mathrm{OM}\;-\;0.20892\mathrm{TN}\;(R^{2}=0.024);$$

$$\mathrm{BSFL}\;\mathrm{DW}=\;-3.15191+0.09675\mathrm{OM}+0.84253\mathrm{SpA}\;\left(R^{2}=0.586\right).$$

In Experiment 1, R^2^ increased to 0.822 for CP and 0.749 for DW, whereas in Experiment 2, R^2^ improved to 0.556 for CP and 0.586 for DW. The regression models were subjected to a series of diagnostic checks to validate their reliability. Residual plots (Figure S3) showed that residuals were randomly distributed around zero with no obvious funnel-shaped patterns, confirming acceptable linearity and homoscedasticity. Variance inflation factors (VIFs) for all predictors were below 2 (Table S3), indicating no significant multicollinearity. Q–Q plots (Figure S4) and Shapiro–Wilk tests (*p* > 0.05 for all models) confirmed that residuals did not deviate significantly from normality. The standard errors (SEs) for all regression coefficients are provided in Table S2, offering uncertainty estimates for the reported R^2^ values The applicable conditions of the models are defined by the experimental data ranges: OM content of 40–50 g, SpA content of 2–6 g, and the use of common agricultural by-products under controlled rearing conditions (4-day-old larvae, 27.5 °C, 70% relative humidity). Predictions are most reliable within these calibrated boundaries.

**Figure 4:**
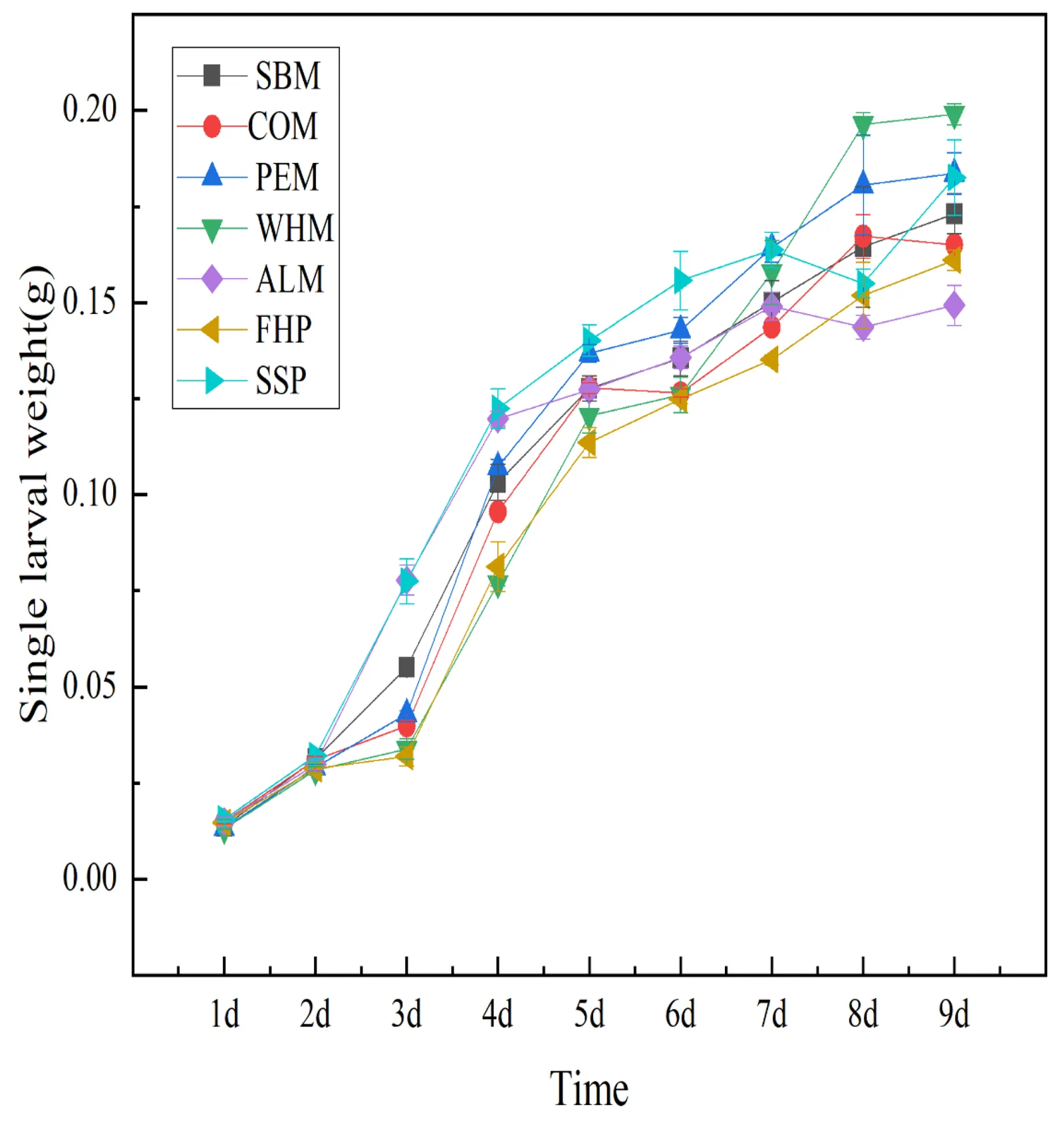
Larval growth performance under standardized SpA intake. Error bars represent means ± SDs of three biological replicates (*n* = 3).

To further validate the role of SpA, Experiment 3 standardized SpA intake across all dietary treatments. This design effectively isolated SpA from other nutritional fractions, confirming its predominant role in larval protein biosynthesis. When SpA intake was equalized (Figure 4), the differences in larval CP and DW among most groups were markedly reduced compared with those under unequal SpA intake (Figure 2A). Across the seven dietary treatments (Table 3), larval CP ranged from 1.36 ± 0.1 g to 2.36 ± 0.2 g, and DW ranged from 3.24 ± 0.1 g to 5.68 ± 0.3 g. No significant differences in larval CP were observed among COM (2.11 ± 0.1 g), WHM (2.36 ± 0.2 g), SBM (2.31 ± 0.1 g), PEM (2.21 ± 0.0 g), and SSP (2.11 ± 0.2 g) (*p* > 0.05). However, ALM (1.36 ± 0.1 g) and FHP (1.74 ± 0.2 g) exhibited lower CP compared with the other groups (*p* < 0.05).

## 4. Discussion

### 4.1. Alkali-Soluble Protein as a Key Determinant of Larval Protein Biosynthesis

**Figure 5:**
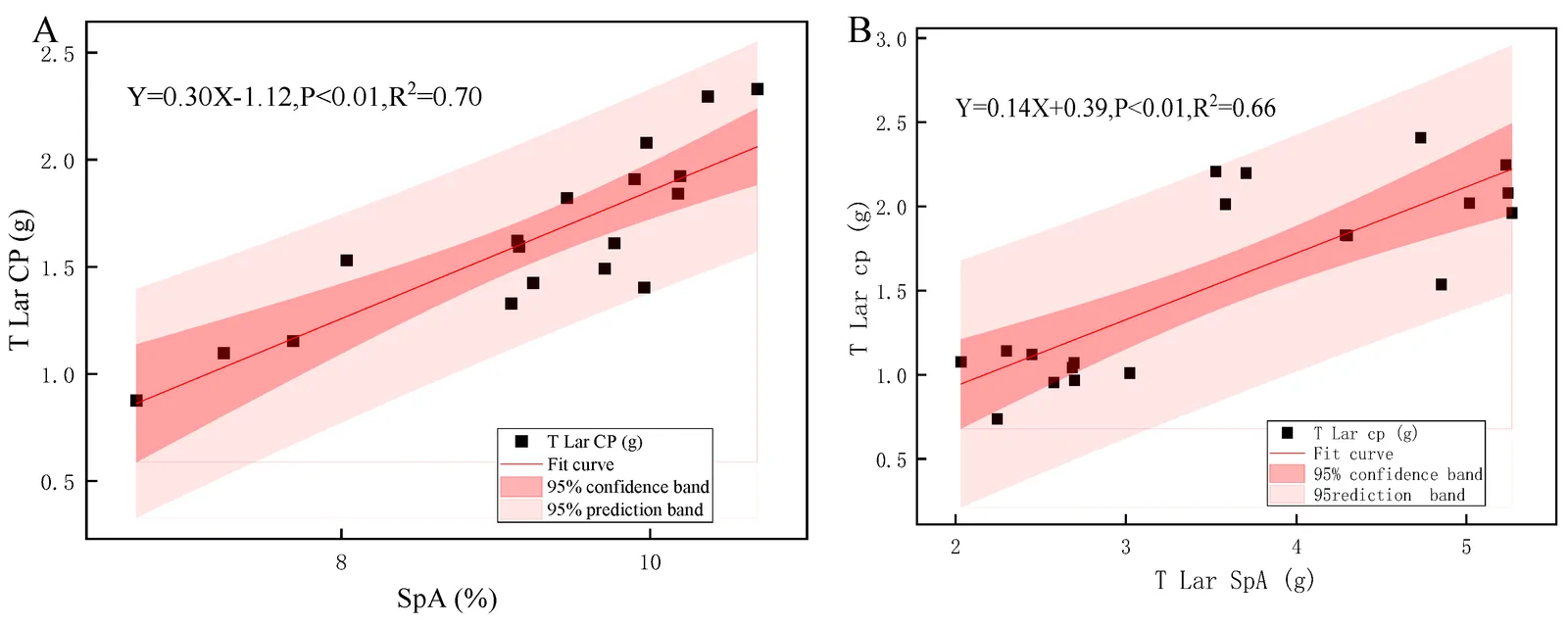
Linear regression between SpA consumption and larval crude protein in Experiment 1 (**A**) and Experiment 2 (**B**).

Dietary alkali-soluble protein is a key determinant of protein accumulation in black soldier fly larvae. The central finding of this study is that SpA serves as a superior predictor of BSFL protein accumulation compared with TN. The correlation heatmap (Figure 1C) revealed that SpA exhibited the strongest positive correlation with larval CP among all measured feed parameters (R^2^ = 0.82), outperforming TN (R^2^ = 0.40). This pattern was further validated in Experiment 2 (Figure 2C), where SpA consistently showed the strongest correlation with larval growth indicators. Linear regression analysis further confirmed a robust positive association between dietary SpA and larval CP in both Experiment 1 and Experiment 2 (Figure 5A,B; R^2^ = 0.85 and 0.82, respectively). These results demonstrate that the bioavailable protein fraction, rather than total nitrogen, is the critical limiting factor for protein biosynthesis in BSFL. This finding challenges the conventional reliance on TN as the primary indicator of feed nutritional quality, a practice that may overestimate or misrepresent the true protein availability for insect growth [27,28].

The superior predictive power of SpA can be attributed to its unique physicochemical properties. Unlike SpC or SpW, SpA fractions are extracted under alkaline conditions (0.2% KOH), which likely select for proteins that are more readily digestible in the BSFL digestive system. SpA, due to its high digestibility, likely facilitates rapid nitrogen assimilation and directs resources toward protein biosynthesis [29,30]. The BSFL midgut maintains an alkaline environment (pH ~ 9–11) [31], which enhances the solubility and bioavailability of SpA while simultaneously creating a favorable niche for alkaline-tolerant, proteolytic bacteria. This host–microbe synergy is likely why SpA, but not other protein fractions, exhibited the strongest correlation with larval growth performance (Figure 1C, Figure 2C and Figure 5A,B). This interpretation is consistent with the report by Laganaro et al. [32], who found that substrate protein digestibility had a stronger influence on BSFL biomass production than total protein content.

Furthermore, the regression models developed in this study provide a quantitative framework for feed formulation. Replacing TN with SpA as the independent variable substantially improved the model’s coefficient of determination for CP from R^2^ = 0.40 to 0.82 (Experiment 1) and from 0.091 to 0.556 (Experiment 2). This improvement is not merely statistical but has practical implications: feed manufacturers can now use the SpA content as a more reliable metric for predicting larval protein yields. The convergence of larval protein content when SpA intake was standardized across divergent feed substrates (Experiment 3; Figure 4; Table 3) provides strong experimental support for a primary limiting role of SpA, beyond a purely observational correlation. This result confirms that SpA availability is the predominant nutritional factor associated with larval protein accumulation.

However, several exceptions require attention. Even under equal SpA intake, larvae fed FHP and ALM consistently exhibited lower CP and DW than those fed other substrates (Figure 4; Table 3). We attribute this to the high fiber content (Table S1), which may physically encapsulate SpA and reduce its accessibility. This phenomenon highlights the importance of fiber content as a confounding factor that can limit the effectiveness of SpA-based feed formulations [33]. Future studies should investigate pre-treatment methods (e.g., enzymatic degradation, fermentation) to reduce fiber interference in high-fiber feedstocks. Beyond fiber, other compositional factors inherent to these agricultural by-products, such as variations in amino acid profiles, anti-nutritional factors (e.g., tannins, phytic acid), and mineral content, may also contribute to the observed differences in larval performance and cannot be completely excluded as residual confounders [34]. Future studies employing purified or semi-purified diets or targeted pre-treatment strategies (e.g., enzymatic degradation of fiber, supplementation of specific amino acids or minerals), will be necessary to isolate the independent effect of SpA from these co-varying factors.

### 4.2. Gut Microbiota as a Functional Mediator of SpA Utilization

**Figure 6:**
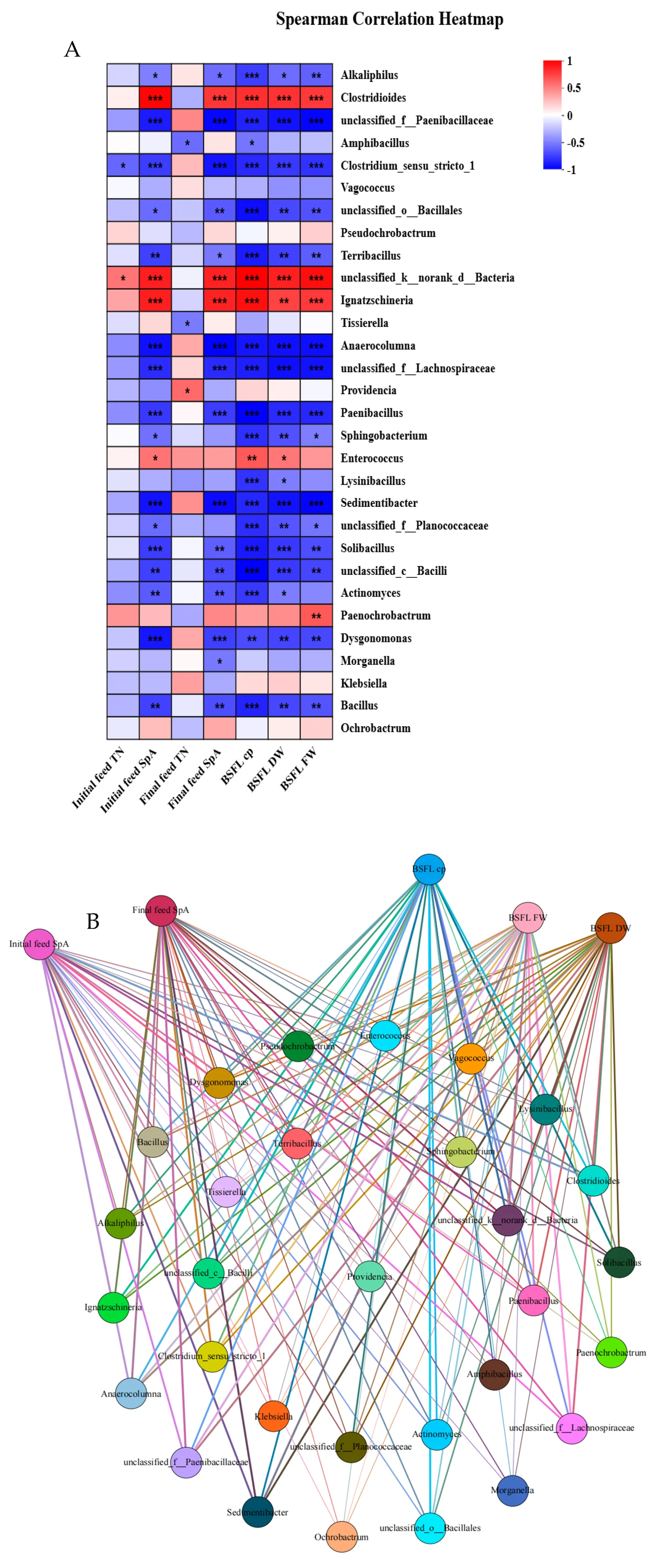
Correlation-based associations between gut microbial genera and larval nutritional parameters. (**A**) The correlations heatmap of larval gut microbial communities on the genus level and their growth conditions. (**B**) The network diagram of larval gut microbial communities on the genus level and their growth conditions. Each node represents a variable (bacterial genus or nutritional factor), and edges indicate interactions. Key network topology parameters are as follows: number of nodes = 35, number of edges = 150, average degree = 8.57, average path length = 1.97, network diameter = 4, clustering coefficient = 0.507, graph density = 0.252. * *p* < 0.05, ** *p* < 0.01, *** *p* < 0.001.

The gut microbiota serves as a functional mediator of SpA utilization in BSFL [35]. Our 16S rRNA sequencing results revealed that high-SpA diets (COM, WHM) selectively enriched specific bacterial genera, most notably *Enterococcus* and *Ignatzschineria*, which were strongly positively correlated with larval CP and DW (Figure 6A,B) [36,37]. By contrast, high-fiber diets (ALM, SSP) promoted the proliferation of *Actinomyce* and *Dysgonomonas*—taxa associated with lignocellulose degradation but negatively correlated with protein accumulation.

The Shannon indices of high-fiber groups (SSP: 2.6 ± 0.1; ALM: 2.3 ± 0.1) were significantly higher than those in high-protein groups (COM: 1.1 ± 0.1; WHM: 1.3 ± 0.1) [38]. The elevated diversity in SSP and ALM groups can be attributed to (1) complex lignocellulose matrices in ALM requiring diverse degradative consortia; (2) nitrogen scarcity under high C/N ratios (SSP: 13.7; ALM: 11.8), which promotes niche partitioning; and (3) energy diversion toward microbial proliferation, reducing larval biomass yields compared with high-SpA diets [39,40]. These patterns are consistent with previous reports [41].

The enrichment of *Enterococcus* in high-SpA groups is particularly noteworthy. Members of this genus are widely reported as core proteolytic bacteria in the insect gut, capable of secreting extracellular proteases [42,43]. We propose the testable hypothesis that *Enterococcus* contributes directly to SpA hydrolysis, releasing smaller peptides and amino acids that are may be absorbed by the larval midgut. The alkaline gut environment likely facilitates the activity of these proteases, creating a positive feedback loop: SpA promotes *Enterococcus* proliferation, which in turn enhances SpA digestion. This symbiotic relationship is supported by the strong positive correlation between *Enterococcus* abundance and larval CP (R^2^ = 0.85), as well as the increased predicted abundance of serine-type carboxypeptidases in the metagenomic profiles of high-SpA groups. However, we acknowledge that this mechanistic inference is currently based on correlational evidence and PICRUSt2-predicted functions and requires direct experimental validation in future studies.

The role of *Ignatzschineria* is more intriguing. This genus has been previously identified in chitin-rich environments such as fly larval guts, with genomes predicted to encode chitinases [44,45]. While not directly involved in proteolysis, *Ignatzschineria* may contribute to gut environment remodeling by degrading chitin in the peritrophic matrix, potentially enhancing nutrient accessibility. We propose that this indirect role, consistent with our observation that *Ignatzschineria* abundance was highest in the WHM group (44%), represents a testable hypothesis that warrants further investigation. The network analysis (Figure 6B) further supports this interpretation, showing co-occurrence patterns among SpA, *Enterococcus*, *Ignatzschineria*, and larval protein, suggesting a coordinated functional module.

**Figure 7:**
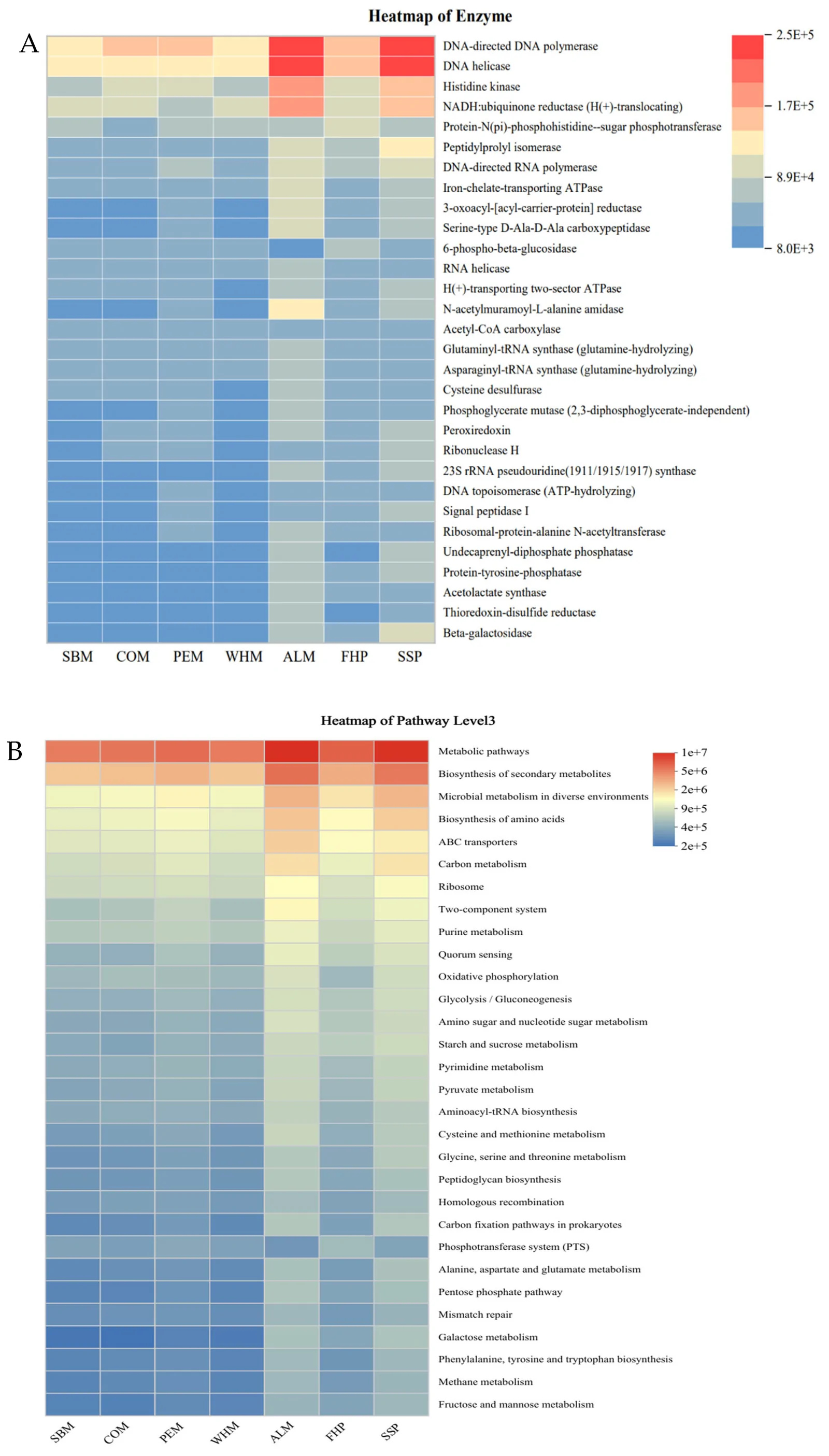
Functional prediction of the BSFL gut microbiota based on PICRUSt2 analysis. (**A**) Heatmap of the predicted relative abundance of key enzyme classes across different dietary groups. (**B**) Heatmap of the predicted relative abundance of KEGG pathways at Level 3. The color gradient from blue to red indicates low to high predicted abundance.

The PICRUSt2-predicted metagenomic profiles (Figure 7A,B) further elucidated the functional basis of the observed microbial community restructuring. In high-SpA groups, the gut microbiota exhibited a higher predicted abundance of serine-type carboxypeptidases and other proteolytic enzymes, consistent with the enrichment of proteolytic taxa such as *Enterococcus*. By contrast, low-SpA groups showed increased predicted abundances of enzymes involved in amino sugar and nucleotide sugar metabolism, alongside the upregulation of peptidoglycan biosynthesis pathways (Figure 7B). This pattern is indicative of a comprehensive “nutrient-stress” response, where the microbial community mobilizes to scavenge resources under SpA limitation [41]. These functional predictions support the hypothesis that SpA availability determines not only the taxonomic composition but also the metabolic programming of the gut microbiota.

By contrast, fiber-degrading taxa such as *Actinomyces* and *Dysgonomonas* showed a negative correlation with CP (R^2^ = −0.76), likely due to nitrogen immobilization during sugar-driven fermentation [46]. This functional trade-off—between host-directed protein synthesis and microbial nitrogen sequestration—explains why high-fiber, low-SpA diets resulted in lower larval protein yields despite comparable TN levels.

### 4.3. The Substrate–SpA–Microbiota Co-Regulation Framework

Based on our integrated experimental and correlational evidence, we propose a “Substrate–SpA–Microbiota” co-regulation model as a testable mechanistic hypothesis for BSFL protein conversion. This tripartite model consists of three interconnected components:

First, dietary SpA content serves as the primary nutritional input. SpA-rich substrates (such as COM and WHM) provide readily available protein that supports rapid larval growth and protein deposition. The isolation effect of SpA was further validated in Experiment 3, in which standardizing SpA intake across divergent feedstocks largely eliminated performance differences among most groups (Table 3, Figure 4), confirming that SpA—rather than other co-varying nutrients—is the primary driver of protein accumulation. The high bioavailability of SpA is attributable to both its physicochemical properties and the alkaline digestive environment of BSFL. This finding aligns with the broader principle that the nutrient form often matters more than nutrient quantity in insect nutrition.

Second, the gut microbiota functions as the functional mediator. SpA enrichment selectively promotes proteolytic taxa (*Enterococcus*) and gut-remodeling taxa (*Ignatzschineria*), which synergistically enhance protein assimilation. *Enterococcus* contributes to extracellular proteolysis, while *Ignatzschineria* may remodel the gut environment to increase nutrient accessibility (Figure 6A,B and Figure 7A,B). By contrast, high-fiber diets shift the microbial community toward carbohydrate degraders that compete with the host for nitrogen, reducing protein conversion efficiency.

Third, the host (BSFL) provides the biological context that enables this co-regulation. The alkaline midgut pH, the extensive repertoire of host-encoded proteases, and the physiological capacity for rapid protein synthesis collectively create an environment where SpA and its associated microbiota can act synergistically. This host context explains why SpA has such a pronounced effect on BSFL protein accumulation compared with other insects or animal systems.

Additionally, the C/N ratio emerged as a significant factor influencing BSFL growth. Optimal growth occurred when the feed C/N ratio was maintained between 5.6 and 10.3, while excessively high C/N values (SSP: 14.7; FHP: 16.2) impaired growth performance. This finding suggests that the SpA content and C/N ratio should be optimized jointly for maximum protein yields—a promising avenue for future multi-objective optimization studies [40].

This framework has implications for sustainable protein production. By selecting feed ingredients with a high SpA content and low fiber levels (e.g., COM, WHM), producers can maximize larval protein yields without relying on expensive protein supplements. The quantitative regression models developed in this study (R^2^ = 0.822 for CP with SpA; Figure 5A,B) provide a preliminary predictive tool for feed formulations, one that could inform future precision nutrition strategies and optimize both economic and environmental outcomes.

We acknowledge that most evidence presented in this study is correlational in nature. While Experiment 3 provides stronger experimental support for the role of SpA, the functional roles inferred for specific gut taxa (e.g., *Enterococcus*, *Ignatzschineria*) are based on 16S rRNA sequencing and PICRUSt2 predictions, which cannot directly measure metabolic activity. The proposed “Substrate–SpA–Microbiota” co-regulation framework should therefore be considered a working hypothesis that requires functional validation. Definitive causal validation will require targeted experiments, including the following: (1) graded supplementation of purified SpA in isonitrogenous and isocaloric background diets; (2) quantitative measurement of intestinal alkaline protease and peptidase activities in vivo; (3) mono- or dual-colonization of germ-free BSFL with candidate taxa such as *Enterococcus* and *Ignatzschineria*; (4) metatranscriptomic or metaproteomic profiling to directly quantify microbial functional gene expression; and (5) pilot-scale rearing trials to validate model predictions under production conditions.

## 5. Conclusions

This study highlights the prominent role of SpA in regulating protein conversion efficiency in BSFL and clarifies the synergistic interactions among feed composition, the gut microbiota, and host metabolism. Our core innovation lies in establishing a “Substrate–SpA–Microbiota” co-regulation framework that shifts the research focus from total nitrogen to the bioavailable SpA fraction. SpA-rich substrates such as COM and WHM markedly enhanced larval protein accumulation, with strong linear correlations between SpA intake and protein biosynthesis (R^2^ = 0.82–0.85). Gut microbiota analysis further identified *Enterococcus* and *Ignatzschineria* as positive contributors to crude protein synthesis, supporting the concept that SpA-enriched diets promote larval protein yields by shaping functional microbial communities. These findings provide a theoretical framework for optimizing protein production from organic waste using BSFL and highlight directions for future research on feed formulation strategies to advance sustainable food systems. Future studies should elucidate the mechanistic metabolic pathways underpinning SpA utilization and evaluate the use of probiotics to further optimize insect-based bioconversion efficiency.

## Figures and Tables

**Table 1:** BSFL nutritional indicators and feed physicochemical properties under different wheat middling/alfalfa meal ratios (Experiment 1).

	Group	Group A	Group B	Group C	Group D	Group E	Group F
	Parameters	5:0	4:1	3:2	2:3	1:4	0:5
BSFL	Crude protein (g)	2.24 ± 0.1 ^a^	1.86 ± 0.0 ^b^	1.64 ± 0.1 ^bc^	1.50 ± 0.0 ^bcd^	1.36 ± 0.1 ^cd^	1.17 ± 0.1 ^d^
	Dry weight (g)	4.2 ± 0.1 ^a^	3.8 ± 0.1 ^b^	3.3 ± 0.2 ^c^	3.0 ± 0.0 ^cd^	2.7 ± 0.1 ^d^	2.1 ± 0.1 ^e^
	Fresh weight (g)	16.0 ± 0.4 ^a^	14.0 ± 0.8 ^ab^	12.9 ± 0.8 ^b^	12.5 ± 0.1 ^bc^	10.6 ± 0.0 ^cd^	8.8 ± 0.5 ^d^
Initial feed	C/N	6.8 ± 0.5 ^a^	7.9 ± 0.3 ^a^	7.8 ± 0.5 ^a^	8.3 ± 0.1 ^a^	7.6 ± 0.1 ^a^	8.0 ± 0.7 ^a^
	TP	0.86 ± 0.2 ^a^	0.80 ± 0.0 ^ab^	0.86 ± 0.0 ^a^	0.64 ± 0.1 ^b^	0.52 ± 0.0 ^b^	0.11 ± 0.0 ^c^
	TK	4.9 ± 0.1 ^a^	5.0 ± 1.2 ^a^	5.9 ± 2.5 ^a^	21.7 ± 2.0 ^b^	4.4 ± 1.1 ^a^	8.3 ± 0.6 ^a^
	SpW	3.3 ± 0.1 ^ab^	3.4 ± 0.1 ^a^	3.1 ± 0.1 ^abc^	2.7 ± 0.3 ^c^	2.8 ± 0.1 ^bc^	3.0 ± 0.1 ^abc^
	SpC	1.3 ± 0.2 ^a^	1.4 ± 0.3 ^a^	1.3 ± 0.0 ^a^	1.3 ± 0.1 ^a^	1.5 ± 0.1 ^a^	1.5 ± 0.1 ^a^
	SpP	14.69 ± 0.6 ^ab^	12.89 ± 1.7 ^b^	17.99 ± 0.9 ^a^	14.55 ± 1.2 ^ab^	10.57 ± 1.4 ^b^	14.56 ± 1.8 ^ab^
	SpA	10.3 ± 0.3 ^a^	9.9 ± 0.4 ^ab^	9.8 ± 0.1 ^ab^	9.4 ± 0.2 ^bc^	8.6 ± 0.2 ^c^	7.3 ± 0.1 ^d^
Finish feed	TP	1.91 ± 0.3 ^a^	1.73 ± 0.1 ^ab^	1.23 ± 0.1 ^bc^	1.07 ± 0.0 ^bc^	1.47 ± 0.2 ^abc^	0.94 ± 0.1 ^c^
	TK	9.0 ± 0.1 ^a^	11.0 ± 2.0 ^a^	8.1 ± 1.6 ^a^	9.9 ± 3.7 ^a^	10.9 ± 0.6 ^a^	10.0 ± 0.3 ^a^
	SpW	2.1 ± 0.1 ^a^	1.0 ± 0.3 ^b^	0.2 ± 0.0 ^c^	0.2 ± 0.1 ^c^	0.4 ± 0.2 ^c^	0.2 ± 0.0 ^c^
	SpC	2.3 ± 0.0 ^a^	1.8 ± 0.1 ^b^	1.4 ± 0.2 ^c^	0.9 ± 0.0 ^d^	0.5 ± 0.1 ^e^	0.4 ± 0.2 ^e^
	SpP	15.25 ± 1.8 ^a^	8.68 ± 2.3 ^a^	11.06 ± 2.6 ^a^	8.39 ± 1.6 ^a^	13.86 ± 1.9 ^a^	10.80 ± 2.4 ^a^
	SpA	0.6 ± 0.1 ^a^	0.5 ± 0.1 ^ab^	0.4 ± 0.1 ^b^	0.3 ± 0.1 ^b^	0.3 ± 0.0 ^b^	0.3 ± 0.1 ^b^

Note: Data are presented as the mean ± SD, *n* = 3 separate rearing containers per treatment. Statistical analyses were performed based on rows and columns, and values in the same row with different superscript lowercase letters were significantly different, *p* < 0.05.

**Table 2:** BSFL nutritional indicators and feed physicochemical properties under unequal SpA intake with different substrate types (Experiment 2).

	Parameters	SBM	COM	PEM	WHM	ALM	FHP	SSP
Unequal SpA								
BSFL	Crude protein (g)	2.05 ± 0.1 ^bc^	2.34 ± 0.1 ^cd^	1.92 ± 0.1 ^b^	2.39 ± 0.1 ^d^	1.06 ± 0.0 ^a^	1.27 ± 0.0 ^a^	1.03 ± 0.1 ^a^
	Dry weight (g)	4.7 ± 0.1 ^a^	4.7 ± 0.3 ^a^	4.7 ± 0.0 ^a^	5.1 ± 0.4 ^a^	2.9 ± 0.1 ^b^	2.7 ± 0.1 ^b^	2.6 ± 0.0 ^b^
	Fresh weight (g)	15.1 ± 0.2 ^a^	16.1 ± 1.0 ^a^	15.6 ± 0.2 ^a^	16 ± 1.5 ^a^	12.5 ± 0.3 ^b^	10.9 ± 0.3 ^b^	11.3 ± 0.1 ^b^
Feed	Feed consumption (g)	36.2 ± 1.3 ^a^	29.7 ± 0.3 ^c^	29.7 ± 0.2 ^c^	33.7 ± 0.7 ^b^	28.2 ± 0.1 ^c^	20.2 ± 0.0 ^d^	25.9 ± 0.6 e
	SpA consumption (g)	5.1 ± 0.1 ^a^	5.1 ± 0.1 ^a^	4.5 ± 0.1 ^b^	3.6 ± 0.1 ^c^	2.8 ± 0.1 ^d^	2.5 ± 0.1 ^d^	2.3 ± 0.2 ^d^
	C/N	11.0 ± 1.1 ^ab^	7.1 ± 1.9 a	11.4 ± 1.8 ^ab^	11.2 ± 1.3 ^ab^	11.8 ± 0.8 ^ab^	14.3 ± 0.9 ^b^	13.7 ± 0.2 ^b^
	Initial feed TN (%)	4.3 ± 0.6 ^a^	4.6 ± 0.1 ^a^	4.5 ± 0.6 ^a^	4.7 ± 0.5 ^a^	4.5 ± 0.3 ^a^	3.8 ± 0.1 ^a^	3.8 ± 0.1 ^a^
	Final feed TN (%)	0.2 ± 0.1 ^b^	1.1 ± 0.5 ^ab^	0.3 ± 0.2 ^b^	1.0 ± 0.5 ^ab^	0.4 ± 0.2 ^b^	2.2 ± 0.0 ^a^	1.0 ± 0.5 ^ab^

Unequal SpA refers to the initial condition from Experiment 2, 100 larvae were fed equal amounts (50 g) of different substrates, which naturally contained unequal amounts of SpA. Note: data are presented as the mean ± SD, *n* = 3 separate rearing containers per treatment. Statistical analyses were performed based on rows and columns, and values in the same row with different superscript lowercase letters were significantly different, *p* < 0.05.

**Table 3:** BSFL nutritional indicators and feed physicochemical properties under standardized equal SpA intake (Experiment 3).

	Parameters	SBM	COM	PEM	WHM	ALM	FHP	SSP
Equal SPA								
	Crude fat (g)	1.4 ± 0.1 ^bc^	1.2 ± 0.1 ^cd^	1.7 ± 0.1 ^b^	2.1 ± 0.5 ^a^	0.8 ± 0.1 ^d^	1.0 ± 0.1 ^cd^	1.1 ± 0.1 ^cd^
BSFL	Crude protein (g)	2.31 ± 0.1 ^a^	2.11 ± 0.1 ^ab^	2.21 ± 0.0 ^ab^	2.36 ± 0.2 ^a^	1.36 ± 0.1 ^c^	1.74 ± 0.2 ^bc^	2.11 ± 0.2 ^ab^
	Dry weight (g)	4.29 ± 0.1 ^cd^	4.05 ± 0.0 ^bc^	4.89 ± 0.2 ^c^	5.68 ± 0.3 ^d^	3.47 ± 0.1 ^ab^	3.24 ± 0.1 ^a^	3.84 ± 0.2 ^abc^
	Fresh weight (g)	15.92 ± 0.2 ^bc^	15.89 ± 0.1 ^bc^	16.87 ± 0.4 ^c^	19.64 ± 0.1 ^d^	13.35 ± 0.2 ^a^	14.22 ± 0.2 ^ab^	16.69 ± 0.2 ^c^
Feed	Initial feed C/N	5.61 ± 0.0 ^a^	5.90 ± 0.2 ^a^	6.4 ± 0.0 ^a^	10.34 ± 0.8 ^b^	9.43 ± 1.0 ^b^	20.85 ± 1.0 ^d^	17.58 ± 0.7 ^c^
	Feed consumption (g)	32.14 ± 0.6 ^c^	28.35 ± 0.1 ^b^	28.22 ± 0.3 ^b^	36.02 ± 0.7 ^d^	38.75 ± 0.6 ^e^	24.26 ± 0.1 ^a^	38.06 ± 0.9 ^e^
	SpA consumption (g)	3.01 ± 0.1 ^a^	3.44 ± 0.0 ^b^	3.22 ± 0.1 ^ab^	3.61 ± 0.1 ^bc^	3.49 ± 0.1 ^bc^	4.06 ± 0.1 ^d^	3.86 ± 0.0 ^cd^

Equal SpA refers to the manipulated condition in Experiment 3, where the feeding amount of each substrate was precisely adjusted so that the total intake of SpA was standardized (equal) across all treatment groups. Note: data are presented as the mean ± SD, *n* = 3 separate rearing containers per treatment. Statistical analyses were performed based on rows and columns, and values in the same row with different superscript lowercase letters were significantly different, *p* < 0.05.

## Data Availability

Data will be made available on request.

## Supplementary Materials

The following supporting information can be downloaded at: https://www.mdpi.com/article/10.3390/insects17080856/s1, Figure S1: Composition of the experimental diets; Figure S2: Standard calibration curve for total nitrogen determination. Note: Absorbance values calculated as A_220_ − 2 × A_275_ were measured for ammonium nitrogen standards at concentrations ranging from 0 to 40 μg mL^−1^. Data points represent the mean of three technical replicates; Figure S3: Residual plot for the regression of BSFL crude protein (CP) on organic matter (OM) and alkali-soluble protein (SpA). (a) in Experiment 1 (b) in Experiment 2; Figure S4: Quantile–quantile (Q–Q) plot of residuals from the multiple linear regression models of BSFL crude protein on organic matter (OM) and alkali-soluble protein (SpA) using data from Experiments 1 and 2 (*n* = 39). The solid line represents the theoretical normal distribution; points closely following the line indicate approximate normality of residuals. Shapiro–Wilk test confirmed that residuals did not deviate significantly from normality (*p* > 0.05); Figure S5: Rarefaction curves of gut bacterial communities at the OTU level (Sobs index); Table S1: Feed Physicochemical Properties; Table S2: Multiple linear regression analysis; Table S3: Variance Inflation Factors (VIF) for all regression models; Supplementary Method S1: Detailed DNA extraction protocol.
