# Supplementary material for: Nutrient–Microbiota Co-Regulation of Protein Conversion in Black Soldier Fly Larvae: The Role of Alkali-Soluble Protein and Gut Microbial Communities
Source: Insects. 2026 Aug 17;17(8):856. doi: 10.3390/insects17080856 (PMC13513417; doi:10.3390/insects17080856)
Supplement: Supplementary file 1 [file insects-17-00856-s001.zip › insects-4428793-supplementary.pdf]

Supplementary Method S1. Detailed DNA extraction protocol

Gut samples were homogenized in 1 mL of sterile water using a sterilized grinder. The homogenate was centrifuged at 12,000 × g for 20 min at 4 °C, and the pellet was collected for DNA extraction. The pellet was resuspended in 1.35 mL of CTAB extraction buffer (100 mM Tris-HCl [pH 8.0], 100 mM EDTA-Na_2 [pH 8.0], 100 mM sodium phosphate [pH 8.0], 1.5 M NaCl, 1% CTAB) containing 10 μL of proteinase K (10 mg/mL), and incubated at 37 °C with horizontal shaking at 225 rpm for 30 min. After adding 150 μL of 20% SDS, the mixture was incubated at 65 °C for 2 h with gentle inversion every 15 min. The supernatant was collected by centrifugation at 6,000 × g for 10 min, and the pellet was re-extracted twice with CTAB buffer and 20% SDS. The combined supernatants were extracted with an equal volume of phenol: chloroform: isoamyl alcohol (25:24:1, v/v), followed by chloroform: isoamyl alcohol (24:1, v/v). DNA was precipitated with 0.6 volumes of isopropanol at room temperature for 1 h, washed with 70% ethanol, air-dried, and dissolved in 200 μL of ddH_2O. RNA was digested with RNase A at 37 °C for 15 min.

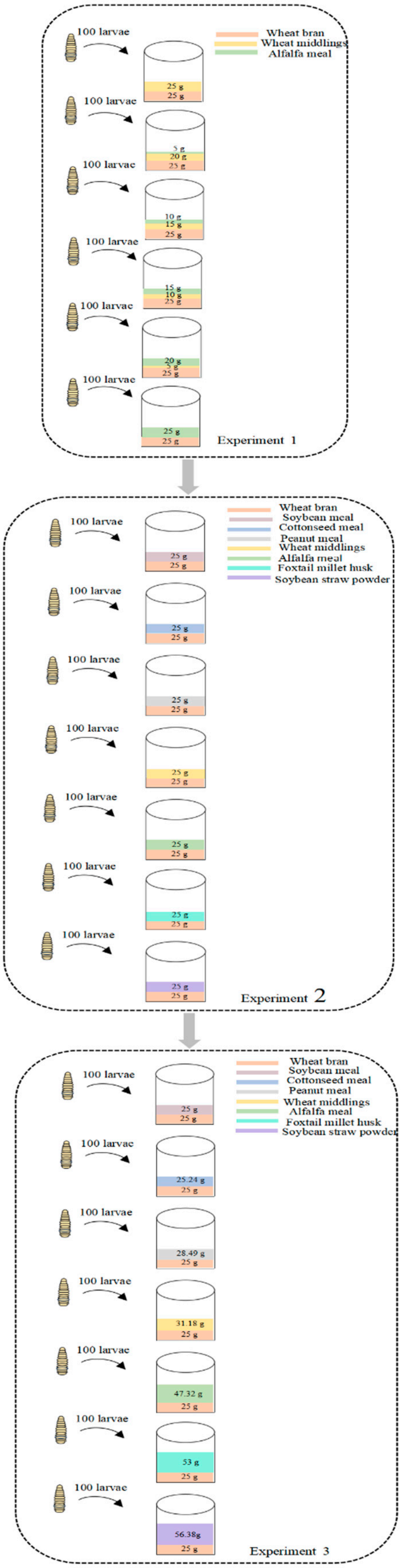

Figure S1. Composition of the experimental diets.

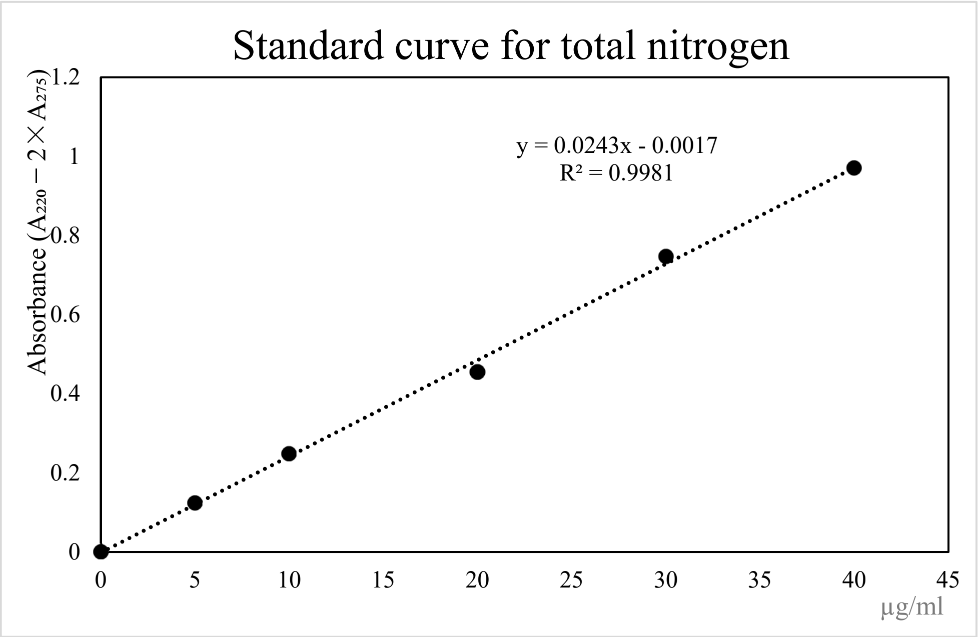

Figure S2. Standard calibration curve for total nitrogen determination. Note**:** Absorbance values calculated as $A_{220} - 2 \times A_{275}$ were measured for ammonium-nitrogen standards at concentrations ranging from 0 to 40 μg mL^{-1}. Data points represent the mean of three technical replicates.

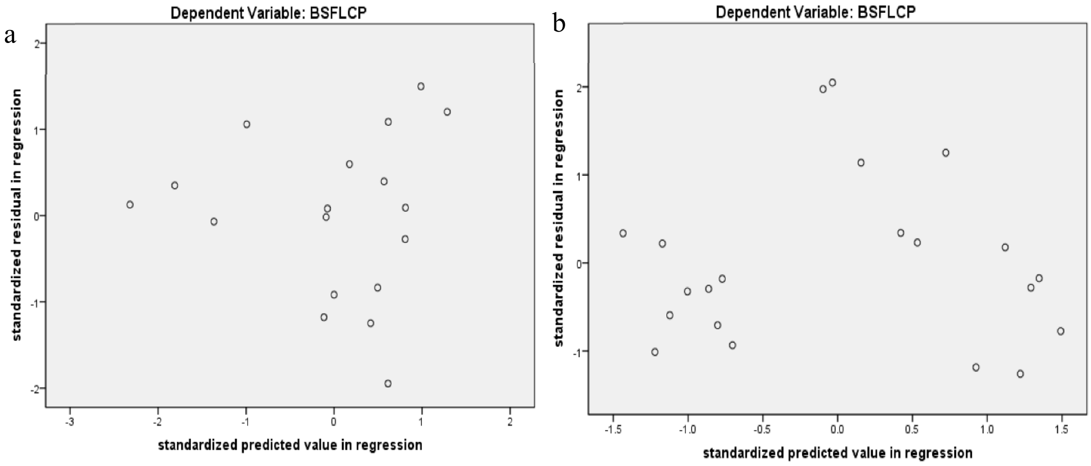

Figure S3. Residual plot for the regression of BSFL crude protein (CP) on organic matter (OM) and alkali-soluble protein (SpA). (a) in Experiment 1 (b) in Experiment 2.

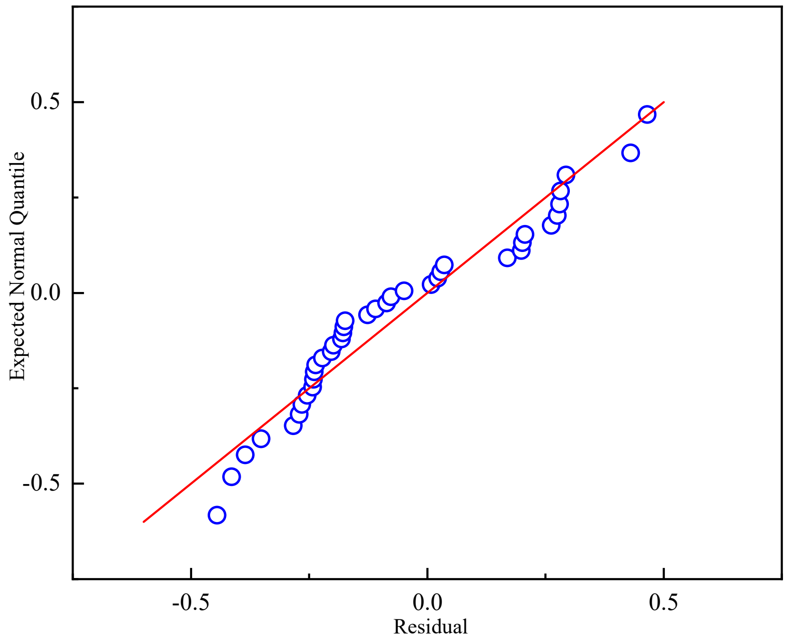

Figure S4. Quantile–quantile (Q–Q) plot of residuals from the multiple linear regression models of BSFL crude protein on organic matter (OM) and alkali-soluble protein (SpA) using data from Experiments 1 and 2 (n = 39). The solid line represents the theoretical normal distribution; points closely following the line indicate approximate normality of residuals. Shapiro–Wilk test confirmed that residuals did not deviate significantly from normality ($P > 0.05$).

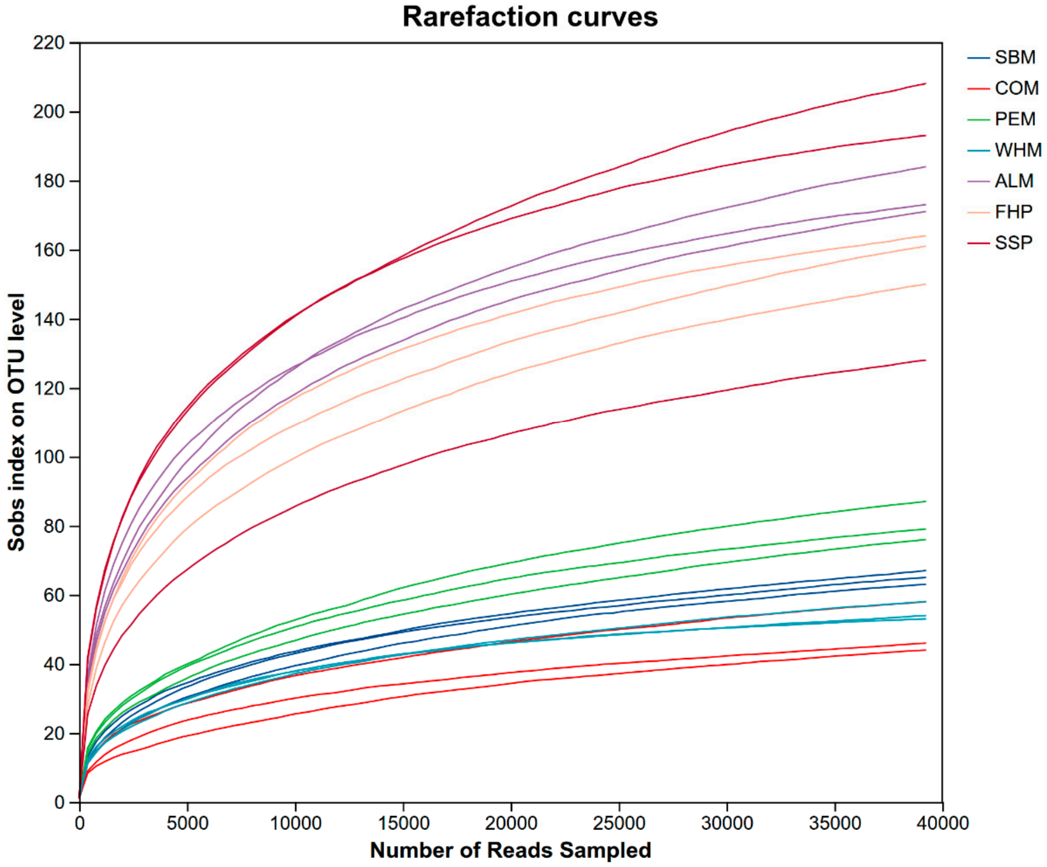

Figure S5. Rarefaction curves of gut bacterial communities at the OTU level (Sobs index). The curves approached saturation for all dietary treatment groups, indicating that sequencing depth was sufficient to capture the majority of bacterial diversity. SBM, soybean meal; COM, cottonseed meal; PEM, peanut meal; WHM, wheat middlings; ALM, alfalfa meal; FHP, foxtail millet husk powder; SSP, soybean straw powder.

Table S1. Feed Physicochemical Properties.

%	wheat bran	wheat middlings	alfalfa meal
TN	6.58±0.60^{b}	5.35±0.90^{a}	8.20±0.8^{c}
TP	3.78±0.31^{a}	3.5±0.02^{a}	3.04±0.22^{b}
TK	5.76±0.02^{a}	6.09±0.13^{b}	5.85±0.05^{a}
OM	21.81±0.03^{a}	23.31±0.01^{c}	22.26±0.02^{b}
SpC	1.50±0.03^{c}	1.30±0.03^{b}	2.74±0.03^{a}
SpW	3.18±0.12^{b}	3.80±0.01^{a}	3.63±0.01^{a}
SpA	9.30±0.32^{c}	10.31±0.30^{b}	6.78±0.36^{a}
SpP	2.03±0.08^{a}	2.1±0.02^{a}	1.65±0.06^{b}
NH_{34}-N	0.013±0.00^{b}	0.021±0.02^{b}	0.76±0.11^{a}
NO_{33}-N	2.39±0.06^{c}	2.09±0.05^{b}	4.38±0.05^{a}

Table S2. Multiple linear regression analysis.

	Dependent variable	Independent variable	Estimate	Standard Error (SE)	P-Value	R^2
Experiment 1	BSFL CP	Constant Parameter	-2.37193	1.09537	-1.22748	0.3961
		OM	0.02294	0.02181	0.5961	
		TN	1.34847	0.22832	3.34793	
		Constant Parameter	-3.81101	0.77662	-3.59667	0.822
		OM	0.04087	0.01494	2.00442	
		SpA	1.12047	0.09558	8.59246	
	BSFL DW	Constant Parameter	-0.3886	1.93235	-0.35477	0.3622
		OM	-0.01353	0.03848	-0.62043	
		TN	0.77848	0.40278	3.40965	
		Constant Parameter	-1.72878	1.05959	-3.44195	0.74909
		OM	0.32163	0.02039	1.96138	
		SpA	0.49201	0.1304	4.90995	
Experiment 2	BSFL CP	Constant Parameter	5.3019	3.14328	1.68674	0.0911
		OM	-0.08451	0.07142	-1.18334	
		TN	0.03117	0.0294	1.06013	
		Constant Parameter	-1.38164	2.66624	-0.5182	0.556
		OM	0.03668	0.05663	0.64773	
		SpA	0.3965	0.0863	4.59425	
	BSFL DW	Constant Parameter	13.31184	6.65606	1.99996	0.02463
		OM	0.01276	0.15123	0.20499	
		TN	-0.20892	0.06225	-1.38152	
		Constant Parameter	-3.15191	5.26286	-0.5989	0.586
		OM	0.09675	0.11178	0.86555	
		SpA	0.84253	0.17035	4.9458	

Note: experiment 1: alfalfa meal at different proportions; experiment 2: different concentrations of SpA.

Table S3. Variance Inflation Factors (VIF) for all regression models.

Experiment	Model No.	Dependent Variable	Predictor 1	Predictor 2	VIF (Predictor 1)	VIF (Predictor 2)	Conclusion
1	1	BSFL CP	OM	TN	1.056	1.056	No multicollinearity
1	2	BSFL CP	OM	SpA	1.004	1.004	No multicollinearity
1	3	BSFL DW	OM	TN	1.056	1.056	No multicollinearity
1	4	BSFL DW	OM	SpA	1.004	1.004	No multicollinearity
2	5	BSFL CP	OM	TN	1.159	1.159	No multicollinearity
2	6	BSFL CP	OM	SpA	1.491	1.491	No multicollinearity
2	7	BSFL DW	OM	TN	1.159	1.159	No multicollinearity
2	8	BSFL DW	OM	SpA	1.491	1.491	No multicollinearity

Notes: VIF, Variance Inflation Factor. A VIF value less than 5 (or more conservatively, 10) indicates no significant multicollinearity.
